# Myelinating glia differentiation is regulated by extracellular matrix elasticity

**DOI:** 10.1038/srep33751

**Published:** 2016-09-20

**Authors:** Mateusz M. Urbanski, Lyle Kingsbury, Daniel Moussouros, Imran Kassim, Saraf Mehjabeen, Navid Paknejad, Carmen V. Melendez-Vasquez

**Affiliations:** 1 Hunter College, Department of Biological Sciences, New York, NY 10065, USA; 2 The Graduate Center, Molecular Cellular and Developmental Biology, The City University of New York, NY 10016, USA; 3 Molecular Cytology Core Facility, Zuckerman Research Center, Memorial Sloan Kettering Cancer Center, New York, NY 10065, USA.

## Abstract

The mechanical properties of living tissues have a significant impact on cell differentiation, but remain unexplored in the context of myelin formation and repair. In the PNS, the extracellular matrix (ECM) incorporates a basal lamina significantly denser than the loosely organized CNS matrix. Inhibition of non-muscle myosin II (NMII) enhances central but impairs peripheral myelination and NMII has been implicated in cellular responses to changes in the elasticity of the ECM. To directly evaluate whether mechanotransduction plays a role in glial cell differentiation, we cultured Schwann cells (SC) and oligodendrocytes (OL) on matrices of variable elastic modulus, mimicking either their native environment or conditions found in injured tissue. We found that a rigid, lesion-like matrix inhibited branching and differentiation of OL in NMII-dependent manner. By contrast, SC developed normally in both soft and stiffer matrices. Although SC differentiation was not significantly affected by changes in matrix stiffness alone, we found that expression of Krox-20 was potentiated on rigid matrices at high laminin concentration. These findings are relevant to the design of biomaterials to promote healing and regeneration in both CNS and PNS, via transplantation of glial progenitors or the implantation of tissue scaffolds.

The cell behavior that drives glial cells to ensheath and myelinate axons depends not only upon cell-cell interactions, but also involves active remodeling of the cytoskeleton. Changes in cell shape are facilitated by intracellular forces generated by actin filaments and myosin motors. These forces are in turn coupled to the ECM via adhesion complexes, which provide anchorage points important for force transmission across the plasma membrane [reviewed in ref. 1]. The concept that mechanical forces regulate cell morphology and differentiation during tissue development is well established^2^ but to our knowledge, it has not been systematically examined in the context of myelinating glial cell development and myelin repair. It is also increasingly recognized that changes both in the chemical and mechanical properties of the ECM play a critical role in various pathologies^3^. Although the nature of the chemical inhibitory signals present in the injured CNS has been extensively characterized and these signals are considered a major impairment to neural regeneration and remyelination^4^,^5^, less is known about the injury-induced changes of the mechanical properties of the extracellular matrix and their impact on tissue repair.

Previously, we identified non-muscle myosin II (NMII) as a key regulator of glial cell differentiation and myelin formation in both the PNS and the CNS^6^. NMII is necessary for the ensheathment of axons by Schwann cells (SC), and its inhibition impairs SC differentiation and myelination^6^,^7^,^8^,^9^. In contrast, inhibition of NMII in oligodendrocytes (OL) promotes cell branching and enhances myelin formation^6^,^7^,^8^. SC and OL undergo striking cytoskeletal changes during differentiation, which influence their distinct patterns of myelination and association with axons. Furthermore, the mechanical properties of the extracellular environments in which SC and OL develop are completely different since in the PNS myelinated axons are surrounded by a basal lamina, which is absent in the CNS.

NMII activity has been previously implicated in regulation of cell morphology and lineage commitment by ECM elasticity^10^,^11^. Thus, soft brain-like matrices (*E *~ 0.5 to 1 kPa) exert neurogenic effects on mesenchymal stem cells, while firmer, muscle-like matrices (*E* ~ 10 kPa) are myogenic, and rigid matrices (*E* ~ 20 to 40 kPa) are osteogenic. Of note, inhibition of NMII abolishes these elasticity-dependent responses^11^. With these precedents in mind, we have tested whether myelinating glial cells might detect and respond to the unique elastic properties of ECM where they develop via NMII-mediated mechanotransduction.

We now report that both OL and SC are capable of responding to the mechanical properties of the matrix, in that stiffer matrices inhibit the branching and elongation of both OL and SC. A more rigid ECM also results in significant reduction in OL branching complexity, which correlates strongly with decreased expression of certain differentiation markers. Thus, changes in the mechanical properties of the CNS following injury may play a prominent role in repair and remyelination. In contrast, SC develop normally in both soft and stiffer matrices, but expression of Krox-20 after cAMP treatment was enhanced in rigid matrices at a high concentration of laminin 2, 1, 1. This supports a biphasic SC differentiation model that integrates both the mechanical and biochemical properties of the ECM and the signals received by the axon.

## Results

### Rigid ECM inhibits OL branching and T3-induced maturation of OPC

**Figure 1:**
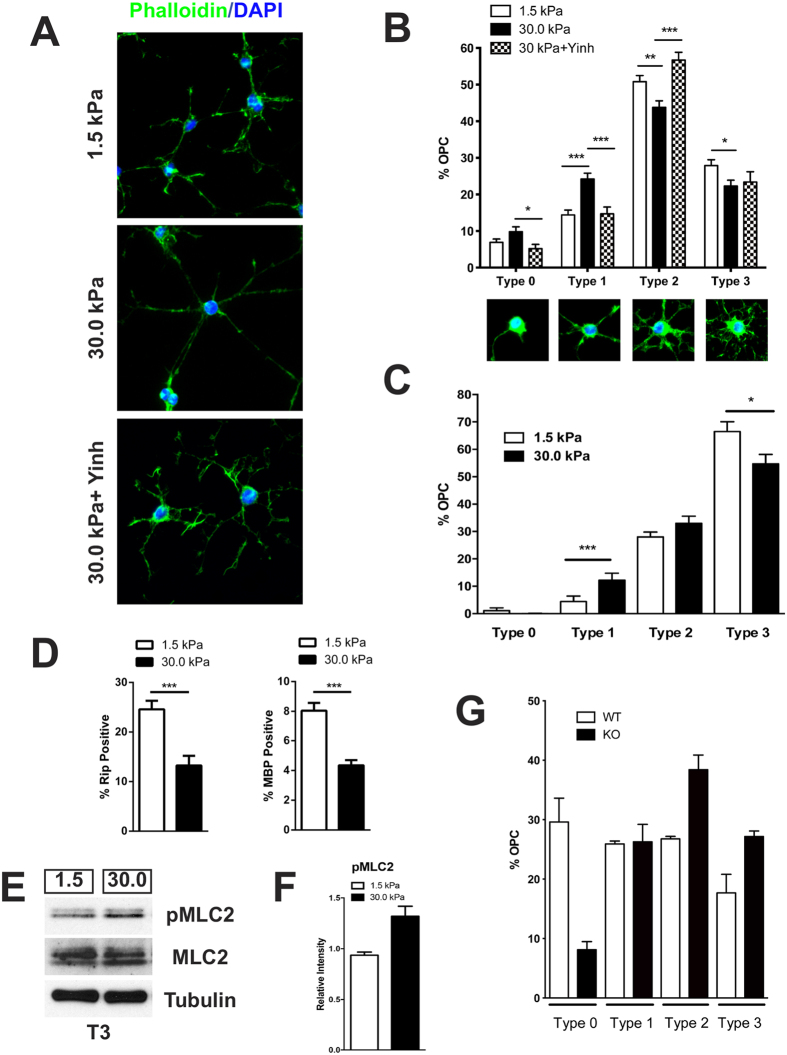
(**A**) OPC cultured for 48 hrs in proliferation-promoting medium (+PDGF) display a significant decrease in branching complexity when grown on rigid substrates (30 kPa) compared to those grown on soft brain-like matrices (1.5 kPa). Inhibition of NMII activity with Y-27632 ROCK inhibitor results in the rescue of OPC branching in cultures grown on rigid substrates (30 kPa + Yinh). (**B**) Quantitation of OL branching complexity in proliferating conditions. Cultures grown on the rigid matrix (black column) display a large increase in the number of cells of low complexity (p < 0.0001) and a significant decrease in the number of high-complexity cells (p = 0.002). Branching is rescued by inhibition of ROCK kinase (checkered column). Approximately 1000 cells were analyzed per condition. (**C**) Quantitation of OL branching complexity in differentiating conditions (+T3) also showed a significant decrease in the number of high complexity Type III cells (p = 0.04), and an increase in Type I low complexity cells (p < 0.0001) in the rigid matrix. Approximately 1000 cells were analyzed per condition. (**D**) OPC cultured on rigid substrates in differentiation-promoting medium (T3) for 48 hours display a decrease in the number of mature RIP+ and MBP+ OL per field (p < 0.0001). (**E,F**) Increased phosphorylation of the regulatory myosin light chain (pMLC2) was detected in OL grown on rigid matrices, indicating higher NMII activity. (**G**) Ablation of NMIIB rescues OPC branching in rigid matrices. OPC derived from NMIIB conditional knockout (KO) mice exhibited increased branching when grown on rigid matrices compared to cells derived from wild type (WT). Data represent mean ± SEM from from 2 independent experiments. On average 170 cells per genotype were analyzed (2 coverslips, 133–229 cells per coverslip/per experiment, Mann-Whitney t-test).

During their differentiation *in vitro*, OPC extend an increasingly complex network of actin-based processes, culminating in the formation of myelin-like sheet lamella. In order to analyze the effects of ECM elasticity on this process, cultures of OPC were seeded on either soft (*E* ~ 1.5 kPa) or rigid (*E* ~ 30.0 kPa) PA/matrigel substrates (Table 1). These elasticity values were chosen as they correspond to previously reported Young’s moduli for the normal brain (range: 0.1–1.6 kPa), and connective/scar tissue (30–40 kPa)^12^,^13^. No significant differences in cell attachment, viability and/or proliferation were observed between the soft and rigid matrices, although we found that for *E* < 1 kPa, OPC attachment and survival were not optimal (data not shown). The value of optimal elasticity for OPC growth found in our studies, partially overlaps with that previously reported using poly-lysine coated matrices^14^,^15^. We found that OL grown on rigid substrates displayed a less complex branching morphology in both proliferating (Fig. 1A,B) and differentiating conditions (Fig. 1C) and expressed lower levels of maturation markers upon stimulation with T3 (Fig. 1D), suggesting that increased ECM rigidity impairs OL differentiation. NMII activity has been shown to regulate the extent of cell branching in response to changes in the elastic properties of the ECM^16^,^17^. We found that OL grown on rigid substrates exhibited a significant increase in levels of phosphorylated myosin light chain (Fig. 1E,F), changes that we have previously demonstrated are directly correlated to inhibition of OL branching and myelination^6^,^8^. Further support for this interpretation was provided by the rescue of OPC branching in rigid matrices in cultures purified from OL-specific NMIIB knockout mice (Fig. 1G)^7^ or by treating the cultures with the ROCK inhibitor Y-27632 (Fig. 1B). Taken together, these results indicate that OPC branching and differentiation was inhibited on rigid lesion-like matrices^12^,^18^,^19^, while softer matrices resembling the mechanical properties of the healthy white matter [reviewed in refs 20 and 21] promoted the process.

### ECM rigidity affects the activity, nuclear localization and levels of Olig1, YAP and lamins in oligodendrocytes

**Figure 2:**
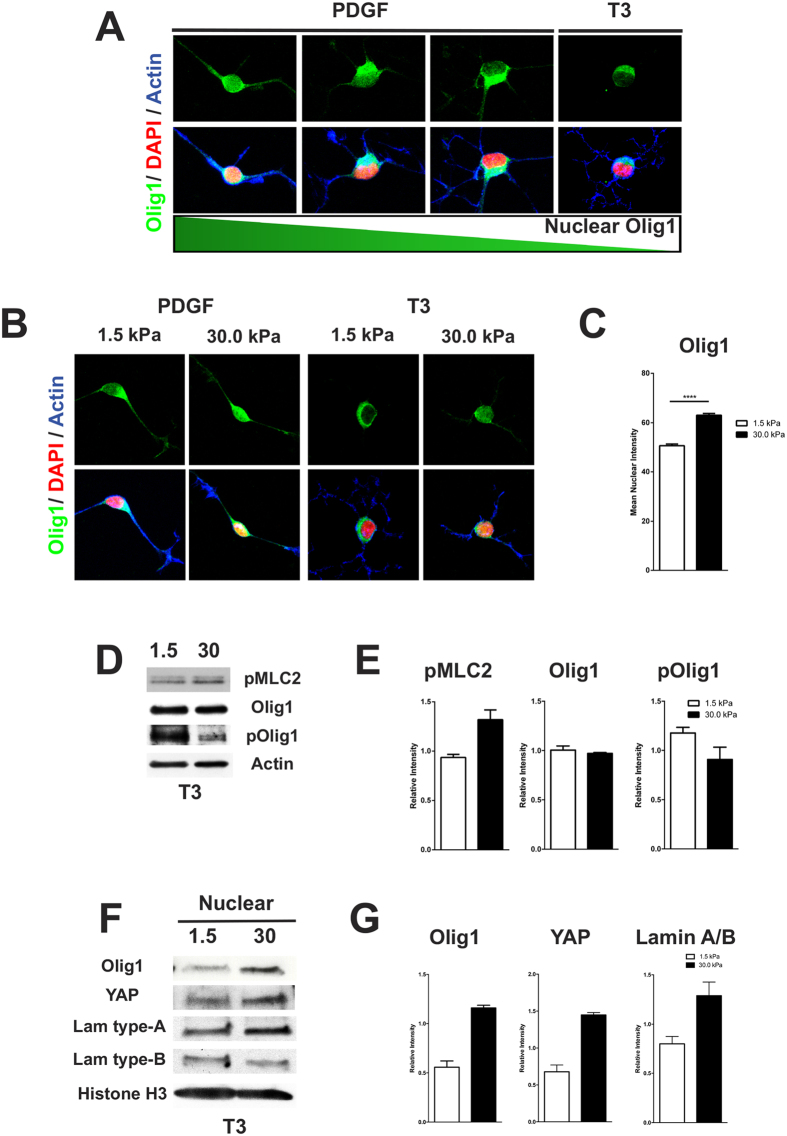
(**A**) Representative images from OPC cultures showing a decrease in nuclear Olig1 and its increased cytoplasmic localization as the cells progress toward a more mature phenotype. (**B,C**) OPC grown on rigid matrices under proliferative (PDGF) or differentiating (T3) conditions display a significant increase in nuclear Olig1 levels (p < 0.0001). (**D,E**) OL grown on softer matrices show decreased phosphorylation of the regulatory myosin light chain (pMLC2), as well as increased Olig1 PKA-dependent phosphorylation. (**F,G**) Increased matrix rigidity also promotes increased levels of YAP and lamin type-A in the nucleus of OPC, while decreasing the levels of lamin type-B. Data on panels C, E and G represents the mean ± SEM from 2–3 independent experiments. Mann-Whitney t-test.

Olig1, a basic helix-loop-helix transcription factor, is a key regulator of OL development^22^. During OL differentiation, Olig1 is phosphorylated and translocated from the nucleus to the cytoplasm (Fig. 2A), an event necessary for membrane expansion and maturation^23^. To gain insights into the possible mechanism connecting the mechanical properties of the matrix with changes in gene transcription and OL differentiation, we investigated whether Olig1 translocation was affected by changes in ECM elasticity. We found that under proliferative (PDGF), or differentiating (T3), conditions OL displayed significantly higher levels of nuclear Olig1 staining when grown on rigid matrices (Fig. 2B,C). Of note, Olig1 cytoplasmic localization depends on its phosphorylation on serine 138 by PKA^23^ and consistent with the observation of increased nuclear levels of Olig1 on rigid matrices, we also found a decrease in phosphorylated Olig1 in these conditions (Fig. 2D,E). In addition, we also found increased nuclear localization of YAP in OL grown on rigid matrices (Fig. 2F,G). YAP is an effector of the mechanosensitive Hippo pathway which is implicated in the control of a variety of developmental processes^24^. Activation of RhoA and high mechanical stress promotes nuclear localization of YAP/TAZ in mesenchymal stem cells (MSC) and drives osteogenesis^24^. In contrast, growth of MSC on a soft surface restricts YAP/TAZ to the cytoplasm and promotes adipogenesis^25^. Recent studies have also found that the ratio of type A to type B lamins in the nuclear lamina is proportional to tissue elasticity. In rigid tissue, the lamina contains predominantly type-A lamins, whereas type-B lamins are prevalent in the lamina of softer tissue^26^. Interestingly, we found that type B lamin expression was reduced, while lamin A was increased in OL grown on rigid matrices (Fig. 2F), thus raising the ratio of type A to type B lamins in comparison to softer brain-like matrices (Fig. 2G). Our results indicated that ECM elasticity can modulate the expression of lamins and the localization of transcription factors known to be important for normal OL differentiation.

### Soft ECM promotes SC elongation and formation of membrane protrusions

We next investigated whether the development of SC was also affected by changes in substrate elasticity. We tested the same elastic modulus value for soft (1.5 kPa) and rigid matrices (30 kPa), as we found that the elasticity of fresh adult sciatic nerve, as measured by AFM nano-indentation, varied between 5–50 kPa and also consistently increases during development (Table 2). Thus 1.5 kPA is still a softer environment compared to that measured for intact and teased fibers; and 30 kPa is within the physiological range for an intact nerve bundle. These data also indicates that the physiological range of peripheral nerve stiffness is very wide. Similar to our findings with OPC cultures, no significant differences in SC attachment or viability were observed between soft and rigid matrices, while a modest but statistically significant increase in proliferation was found on 30 kPa substrates (Supplementary Fig. 1). This increase in cell proliferation on rigid matrices has been previously reported for other cell types^27^.

**Figure 3:**
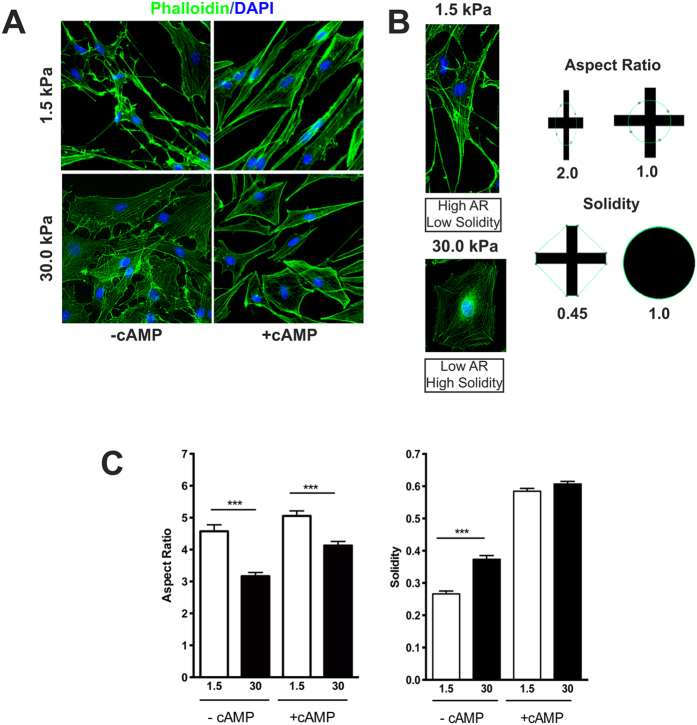
(**A**) SC morphological changes in response to ECM elasticity. SC grown on soft substrates (1.5 kPa) showed greater elongation and process extension, while those grown on rigid substrates (30 kPa) displayed a more polygonal morphology. (**B**) Representative examples of SC displaying the changes in aspect ratio (cell elongation) and solidity (cell branching) observed at different matrix stiffness. (**C**) Quantitation of changes in SC morphology. SC grown on soft matrices exhibited a significantly higher aspect ratio and lower solidity than those grown on rigid substrates. (***p < 0.001). Approximately 400 cells per condition were analyzed (2 coverslips, 100 cells per coverslip/duplicate experiments, Mann-Whitney t-test).

Although we found that SC also responded to variations in ECM elasticity by adopting strikingly different morphologies, the impact of these purely mechanical changes on SC maturation was less significant compared to those exhibited by OL. SC plated on soft matrices appeared elongated and exhibited more actin-rich processes along the membrane, while SC on rigid matrices adopted a flattened morphology and had more stress fibers (Fig. 3A). To quantify these morphological differences, we carried out analyses of the aspect ratio and solidity of SC^28^. Aspect ratio corresponds to cell elongation while solidity is reflective of cell branching. Thus, cells with high aspect ratio and low solidity will be more elongated and have a large number of processes; while more polygonal cells would have higher solidity and a lower aspect ratio (Fig. 3B). We found that, SC grown on softer matrices had a significantly higher aspect ratio and lower solidity when compared to those grown on rigid substrates (Fig. 3C).

### Expression of SC differentiation markers is unaffected by matrix elasticity alone

**Figure 4:**
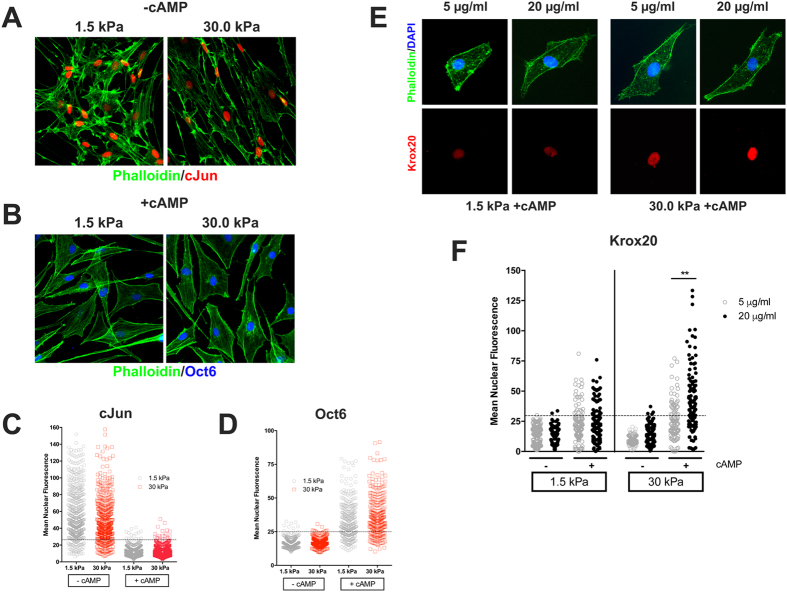
(**A,C**) Undifferentiated SC (-cAMP) growing in either soft or rigid substrates showed comparable levels of cJun expression. Addition of cAMP (+cAMP) down-regulates cJun in both soft and rigid matrices. Dashed line indicates the background level of c-Jun expression (mean + 2 standard deviation) measured in control cultures (cells grown on poly-lysine + cAMP). (**B,D**) Up-regulation of Oct6 expression after cAMP treatment was also observed in both conditions. Dashed line indicates the background level of Oct 6 expression (mean + 2 standard deviation) in measured in control cultures (cells in grown polylysine -cAMP). Approximately 400 cells were analyzed per condition (2 coverslips/100 cells per coverslips/duplicate experiments, Mann-Whitney t-test) (**E,F**) Representative images from cultures stimulated with cAMP showing the effect of matrix stiffness and laminin concentration on Krox 20 expression. (**F**) Upregulation of Krox20 expression after cAMP treatment is potentiated by increased laminin concentration in rigid matrices (p = 0.005). A higher percentage of Krox20 positive cells were found in rigid matrices at the highest laminin concentration. Dashed line indicates the background level of expression (mean + 2 standard deviation) in control conditions (non-stimulated cells grown on poly-lysine). Approximately 100–170 cells were analyzed per condition (2 coverslips/50–70 cells per coverslips/duplicate experiments, Mann-Whitney t-test).

We next investigated whether the expression of transcription factors known to regulate SC differentiation was affected by ECM elasticity. We found that the levels of c-Jun (Fig. 4A), a negative regulator of SC differentiation, and the levels of Oct6 (Fig. 4B), a pro-myelinating transcription factor, were relatively unaffected by ECM stiffness. Although we found that the average mean nuclear fluorescence for c-Jun prior to cAMP treatment, was higher in SC grown on the softer matrices and that of Oct6 was lower on softer matrices (Fig. 4C,D) these differences were small (10–15%) and not detectable by western blot (Supplementary Fig. 2). SC in both soft and rigid matrices responded similarly to cAMP treatment (a signal mimicking the pro-myelinating stimulus from axons) by down-regulating c-Jun and up-regulating Oct6 (Fig. 4C,D) and Krox20 (Supplementary Fig. 2). Addition of cAMP also resulted in SC adopting a similar polygonal morphology regardless of matrix elasticity (Fig. 4B). Of note, although the initial levels of phosphorylated MLC were increased in SC plated on rigid matrices, addition of cAMP abolished these differences in NMII and FAK activation (Supplementary Fig. 2). Collectively, our data indicated that ECM elasticity affects SC morphology but it does not affect their differentiation as measured by cAMP-mediated downregulation of c-Jun and upregulation of pro-myelinating transcription factors (Oct6 and Krox20).

### Increased concentration of laminin 2, 1, 1 in stiffer matrices potentiates cAMP-mediated SC differentiation

Prior to myelination, SC select segments of axons in a 1:1 relationship. The coordination between signaling events occurring at the abaxonal and adaxonal SC membranes is central to this process known as radial sorting. Recent studies have shown that GPR126, a G-protein coupled receptor essential for PNS myelination, has a dual role in this process via its interaction with laminin 2, 1, 1, which provides a mechanistic link between ECM signals and the up-regulation of cAMP production by SC^29^. Of note, GPR126 responds to changes in mechanical force, thus suggesting that it might be a mechanosensitive receptor^29^,^30^. In order to explore whether Krox20 expression by SC in response to ECM-elasticity and cAMP stimulation was modulated by the composition of the basal lamina, we plated SC on soft and rigid matrices prepared with collagen I (0.1 mg/ml) and different concentrations of laminin 2, 1, 1 thus mimicking the composition of an immature (5 μg/ml) or mature (20 μg/ml) basal lamina^31^. We found that after addition of cAMP the up-regulation of Krox20 expression was significantly greater in SC plated on rigid matrices coated with laminin compared to those plated on soft matrices, with the maximum response observed at the higher laminin concentration (Fig. 4E,F). Taken together, our data indicated that high concentration of laminin enhances Krox 20 expression in rigid matrices.

## Discussion

Collectively, our data demonstrated that while myelinating OL and SC carry out a similar function, they accomplish it in a strikingly different manner, informed by the environmental demands of their developmental niches. OL, which myelinate multiple axons and inhabit an environment featuring a loose and compliant ECM, differentiate optimally when experiencing very low mechanical forces and levels of actomyosin contractility. Conversely, as SC develop within firm nerve bundles and secrete their own basal lamina, their differentiation is dependent on sufficient ECM density and promoted by increased mechanical stress.

Increased ECM rigidity results in significant reduction in OL branching complexity, a change that correlates strongly with decreased expression of differentiation markers. We also found that OL grown on rigid substrates exhibited a significant increase in levels of phosphorylated myosin light chain and NMII activity, changes that we have previously demonstrated are directly correlated to inhibition of OL branching and myelination^6^,^8^. Further evidence attesting to a role for NMII-mediated contraction in this process was provided by the rescue of OL branching complexity on rigid matrices following ROCK inhibition and/or genetic ablation of NMII. NMII activity has been shown to regulate the extent of cell branching in response to changes in the elastic properties of the ECM^16^,^17^. Thus, cells growing on soft matrices resembling the mechanical properties of the healthy white matter^20^,^21^ form many cell processes, whereas when cultured on rigid matrices they make fewer branches.

*In vitro* studies have shown that increased matrix stiffness promotes a reactive phenotype in astrocytes and other glia^20^,^32^, and that matrices with high mechanical compliance similar to the normal brain support the growth of neurons without activation of astrocytes^21^,^33^. Although the stiffness of the astrocytic scar has not ben directly measured in human tissue, it is well established that astrocytosis is a prominent feature of the chronic demyelinated MS plaques, an environment where remyelination and repair is impaired^34^,^35^. These changes in tissue structure are accompanied by sclerosing of the lesions, which is comparable with the mechanical strength of other fibrous scars in the human body [reviewed in refs 36 and 37]. Due to this pathological feature, i.e: the presence of multiple hardening plaques, this disease was first named multiple sclerosis. Therefore, it is feasible that at sites of injury where astroglial scarring occurs changes in ECM elasticity might act as a barrier to nerve repair and remyelination^33^.

Of note, in the intensively gliotic, chronically demyelinated lesion in MS, myelin repair, when present, is commonly limited to a narrow ring at the lesion margin abutting normal appearing white matter^38^. Similarly interesting is the observation that more widespread remyelination may be a feature of active, acute lesions in MS, prior to the formation of astrocytic scar^39^. In agreement with this interpretation, magnetic resonance elastography studies in patients with diagnosis of relapsing-remmitting MS found a global decrease of brain viscoelasticity at early disease stage^40^. Although studies directly measuring the stiffness of chronic MS lesion are not available, *“the tissue comprising a chronic MS lesion is quite hard and stiff. The classic Lhermitte signs are electric-like pains in the neck due to hardened lesions in the cervical spinal cord pulling on adjacent spinal nerve roots during neck flexion.”* (Cedric Raine, personal communication. See also ref. 36). Furthermore, ongoing studies in our laboratory have shown that acute demyelination after lysolecithin injection causes a drop in tissue stiffness, while chronic cuprizone demyelination causes an increase in tissue stiffness (Urbanski and Melendez-Vasquez, unpublished data). Therefore, similar to the present paradigm, an increase of the matrix rigidity in chronic MS might have a negative effect upon myelin formation in the CNS. Therefore, inhibition of cytoskeletal tension may offer an additional pathway to overcome the restrictive environment found in chronic CNS lesions. In support of this interpretation, we recently reported that conditional ablation of NMII in OL accelerated the repair of demyelinated CNS lesions^7^.

Current research into mechanisms underlying the transduction of mechanical signals into changes in gene expression have identified pathways that regulate the translocation of transcription factors in/out of the nucleus as well as proteins that vary systematically with changes in ECM elasticity. We found that Olig1 is retained in the nucleus in OL grown on rigid substrates and also exhibits a lower level of phosphorylation, which negatively correlates with OL membrane expansion and maturation^23^. Additionally, in agreement with previous studies^25^, in OL grown on rigid matrices we found increased nuclear localization of YAP, an important transducer of mechanical forces into transcriptional regulatory cues. We have also observed elasticity-dependent expression of nuclear lamins. The nuclear lamina is a network of structural proteins connecting the nuclear envelope to chromatin, and yet another important component in the transmission of signals from the environment into the nucleus. Differences in the expression of lamin A and lamin B might result in changes in chromatin organization and gene transcription thus impacting cell differentiation. Of note, genes required for OL maturation and CNS myelination are affected by abnormal expression of lamin B1 in adult onset autosomal leukodystrophy (ADLD)^41^.

We found that type B lamin expression was reduced, while lamin A was increased in OL grown on rigid matrices, thus raising the ratio of type A to type B lamins in comparison to softer brain-like matrices. This is a very novel observation, and to our knowledge the first demonstration that changes in ECM stiffness affect the ratio of lamin A/B expression in OL, although increases in lamin A/B ratio in response to tissue stiffness have been reported in other cell types^26^. The importance of this observation is further underscored by the recent publication^42^; showing that overexpression of lamin B1 in OL results in demyelination and this defect is linked to changes in lipid synthesis and decreased cholesterol production. Of note, it is well established that membrane cholesterol directly affects the mechanical properties of the cell membrane. Specifically, decreased cholesterol levels results in decreased fluidity and increased membrane stiffness^43^,^44^. Thus in OL changes in the composition of the nuclear lamina might have an impact on the mechanical properties of the plasma membrane.

Although we found that SC also responded to variations in ECM elasticity by adopting strikingly different morphologies, the impact of these purely mechanical changes on SC maturation and differentiation was less significant compared to those exhibited by OL. Thus, treatment with cAMP resulted in upregulation of SC differentiation markers to comparable levels on both soft and rigid collagen-based matrices. Myelinating SC are radially polarized cells^45^, exhibiting two distinct membrane surfaces: one apposing the axolemma (adaxonal surface) and other underlying the basal lamina (abaxonal surface). The basal lamina, a specialization unique to myelinated PNS fibers, is secreted and assembled by SC following axonal envelopment^46^ and is the direct source of ECM-derived signals required for normal PNS myelination^47^. Therefore, it is possible that the direct control of SC over the composition of their own ECM allows them to modulate their responses to mechanical cues, a phenomenon which would not occur in the CNS due to the lack of a basal lamina. In support of this hypothesis, we found that increasing the concentration of laminin 2, 1, 1, the main component of mature basal lamina, potentiates the expression of the pro-myelinating transcription factor Krox-20, following cAMP treatment on rigid matrices. We also found that the elastic modulus of fresh sciatic nerves increases steadily during the first week of development as they become myelinated (Table 2).

Previous work has demonstrated that the maturation state of the basal lamina (immature vs. mature) impacts the function of FAK during SC development^31^. FAK promotes the spreading and proliferation of SC via actomyosin contractility in immature basal lamina, thereby stimulating their proliferation and proper radial sorting. Once radial sorting is accomplished and the basal lamina matures, SC differentiation becomes independent of FAK activation^31^. We also found evidence of a biphasic dependence of SC differentiation on actomyosin contractility, with increased phosphorylation of MLC in SC maintained in undifferentiated conditions, followed by a decrease in actomyosin activity after stimulation with cAMP^9^. Of note, although we found that decrease in actomyosin activity in SC promoted expression of Krox20 and myelin proteins, the cells failed to produce compact myelin when in contact with axons^9^.

Collectively, these results and previous work from our group and others^8^,^9^,^48^,^49^, suggest a model for SC myelination that requires coordination between signals generated at the abaxonal membrane, i.e. laminin engagement and activation of actomyosin tension, and signals in the adaxonal membrane leading to increased cAMP levels and decreased cytoskeletal tension. Together, these signaling events will ultimately promote myelin gene transcription and facilitate membrane expansion and wrapping^9^. The recent discovery that different domains within the mechanosensitive GPR126 are at the center of this spatial and temporal regulation of SC differentiation, and that the maturation status of the basal lamina can in turn modulate the response of SC to GPR126 activation^29^, provides a mechanistic link for the integration of mechanical and biochemical signals in PNS myelination. While this manuscript was under revision a study showing that YAP/TAZ are transiently activated in SC after mechanical stimulation and regulate radial sorting and expression on basal lamina receptor was published^50^, lending further support to this model.

A number of recent studies have examined the feasibility of using implanted biomaterial conduits or engineered tissue grafts in peripheral nerve repair. However, while the impact of the chemical composition of the nerve conduits has been addressed, the potential importance of the mechanical properties of these materials–while acknowledged^51^ –remains unexamined. Similarly, while attempts to design engineered tissue grafts composed of extracellular matrix protein and SC have demonstrated that mechanical forces are important for inducing ECM organization conducive to nerve repair^52^, and other efforts have focused on maximizing nerve repair through providing appropriate chemical cues by seeding the implant with SC engineered to over-express neurotrophic factors^53^, the mechanical properties of these constructs were not investigated with a view toward optimizing both neuronal outgrowth and SC differentiation.

Although, the culture system we developed in this study does not recapitulate the polarization of SC into abaxonal (ECM) and adaxonal (axon) surfaces, it allowed us to evaluate their response to changes in the mechanical properties of the matrix, an aspect that has not been previously explored. Similarly, despite the fact that the global changes in pMLC and pFAK levels in response to stiffness were minor they might still be biologically relevant if they were to be developmentally and/or regionally restricted in a polarized SC. Furthermore, the data showing that changes in ECM ligand density (increased laminin concentration) in combination with a stiffer matrix potentiates the expression of Krox20 demonstrate that this experimental approach can be employed to further explore the combined role of the mechanical and chemical properties of the matrix in SC differentiation. A similar approach for the study of OL maturation was recently published^54^.

## Materials and Methods

### Preparation of glial cultures on ECM protein substrates of varying elasticity

Thin polyacrylamide substrates (PA) of different viscoelastic moduli were prepared as previously described^17^,^55^. Briefly; NaOH-cleaned 12 mm glass coverslips were coated with 3-aminoproyltrimethoxysilane and incubated with 0.5% glutaraldehyde for 30 min at RT. PA solutions ranging from 7.5 acrylamide/0.05% bis (Young’s modulus *E* ~ 1.5 kPa) to 12% acrylamide/0.28% bis (Young’s modulus *E* ~ 30 kPa), were pipetted onto treated coverslips and allowed to polymerize for 10 min at RT. To make a sandwich gel, PA gels were activated for crosslinking with UV using Sulfo-SANPAH (Pierce) and incubated with ECM protein (0.1 mg/ml Collagen I or 0.1 mg/ml Collagen I plus 5–20 μg/ml laminin for PNS cultures; 20 μg/ml matrigel for CNS cultures) overnight at 37 °C. After extensive washing, primary glial cells (3–5 × 10^4^ for IF or 1 × 10^6^ for WB) were plated on top of the crosslinked substrates and allowed to adhere overnight. To verify that the elastic properties of the substrates were not modified by protein coating, similar PA-matrigel soft and rigid gels were by AFM as described below, and compared to uncoated controls. Representative indentation force curves are shown in Supplementary Fig. 3. To verify uniform protein coating, matrigel-coated soft and rigid matrices were immunostained for laminin, imaged by epifluorescent microscopy, and quantified using ImageJ (Supplementary Fig. 3).

### Atomic Force Microscopy (AFM)

The elastic modulus of soft and stiff PA gels was measured by AFM nano-indentation and confirmed to match the predicted values (Table 1). Briefly, an Asylum Research MFP-3D-BIO Atomic Force Microscope was used to collect force curves and force maps of the two groups of gels. A CP CONT-PS C (NanoAndMore.com) probe with a 6.1 μm polystyrene bead was used for the spherical Hertz model indentations, and an Olympus TR400PB Long Cantilever (AsylumResearch.com) was used for the pyramidal Hertz model indentations. The Asylum Research GetReal calibration method was utilized for calibration of the spring constant. We have independently verified that the GetReal calibration gives adequate calibrations when compare directly to the thermal tune method for these probes. The same probe was used for all measurements amongst and between groups, and the groups were rotated between measurements. Force maps were conducted in PBS. Curves were collected to a trigger point of 100 nN for spherical indenters and 25 nN for pyramidal indenters. A 1 second dwell was incorporated into the force curve to elucidate viscous behavior. A 50 μm/s indentation velocity was employed because we found the gels to have a high rate of creep, with slower velocities causing the gel to yield to the bead and preventing clean force curves from being collected. The curves were fit with the Hertz model as utilized in the Asylum Research software. Each force map samples a grid across a 90 μm × 90 μm area. Shallow indentation analyses were restricted to <2 μm depth areas. We sampled 4 gels from each group with the spherical indenter, and each gel was sampled 3 times, producing a total of 12 force maps consisting of 256 curves per sample, or a total of 3072 measurements for each type of gel. For tissue measurements, fresh brain and nerves (teased vs. unteased fibers) were compared, as well as spherical vs. pyramidal indenters. Vertebrate animal tissue (brains and nerves) was collected in accordance with the National Institutes of Health guidelines. All procedures were approved by Hunter College Institutional Animal Care and Use Committee.

### Glial cell purification

A2B5 + oligodendrocyte precursors (OPC) were purified by immunopanning from mixed glial cultures of postnatal day 2 rat cerebral cortices as previously described^6^. Purified OPC were seeded on to PA/ECM elastic substrates and maintained in either proliferation media with PDGF (10 ng/ml) and bFGF (10 ng/ml), or induced to differentiate in media containing T3 (30 ng/ml). For inhibition studies, Y267632 (Calbiochem) was added to the media (10 μM) and maintained throughout the experiment (1–3 days).

Mouse OL were prepared as previously described^56^. Briefly, mixed glial cultures were prepared from postnatal day 2 mouse cerebral cortices of *PlpCre/ESR1*:Rosa26-mT/mG:*NMIIB*^*fl/fl*^ and *PlpCre/ESR1*:Rosa26-mT/mG:*NMIIB*^+/+^ animals^7^, and used to generate OL-enriched glial cultures by separating the OL from the astrocyte monolayer by orbital shaking, followed by purification by differential adhesion to plastic. Purified OPC were seeded on to PA/ECM elastic substrates. Recombination was induced with 1 μM tamoxifen and the cells were allowed to differentiate for 48–72 hours in medium containing T3 (30 ng/ml).

Schwann cells (SC) were isolated from postnatal day 2 rat sciatic nerves and expanded for 3 wk in M media (MEM, 10% FBS and 2 mM L-glutamine), supplemented with 4 μM forskolin (Sigma), and 5 ng/ml of the EGF domain of rhNRG-1-β1 (R&D Systems), hereafter called M^+^ media. For studies on the effects of cAMP analogues on SC differentiation, cultures maintained in M^+^ media were switched to media without growth factors for 3 d (M media). SC were either starved overnight in serum-free M media (MEM and 2 mM L-glutamine), or placed in defined media DM (DMEM/F12, 5 μg/ml insulin, 5 μg/ml transferrin), prior to treatment with 1 mM dibutyryl cyclic adenosine monophosphate (db-cAMP) (Calbiochem), for 24 h, 48 h and 72 h.

### Immunofluorescence

Glial cell cultures were fixed in 4% PFA and processed for immunocytochemistry as previously described^6^. Cultures were examined by epifluorescence using a Leica DMI4000 microscope or a Zeiss LSM 510 confocal microscope. Image analysis (see below), was performed using ImageJ 1.38v and Adobe Photoshop CS8. For quantitation of nuclear fluorescence intensity, random 20X fields were captured from cultures co-stained with DAPI and phalloidin as well as immunostained for either c-Jun, Oct6, Krox-20 or Olig1. The DAPI channel was used to establish areas of interest corresponding to the nuclei by using the ImageJ nucleus counter tool with manual corrections. The ROIs established by the nucleus counter were added to the ROI manager, and then used, along with the ImageJ measure tool, to record the mean gray value of c-Jun, Oct6, Krox-20 or Olig1 staining within the nuclear regions. Background fluorescence was measured in the areas outside the nuclear ROIs, averaged, and subtracted from the mean nuclear gray value.

### Image analysis

OPC were counted in micrographs from 10–12 random low-power fields/coverslip, using ImageJ (total of 20–30 fields per condition per experiment; total of 2–3 experiments). For the evaluation of morphology, OPC were counted and classified according to their branching complexity as follows: (0): no branching; (1) low complexity: cells with at least one or two branches directly from cell body; (2) medium complexity: cells with secondary processes extending from primary branches and (3) high complexity: cells with tertiary processes extending from secondary branches.

The shape of Schwann cells was determined by drawing a best-fit ellipse around the cells using ImageJ (National Institutes of Health), and determining their solidity and aspect ratio. Aspect ratio (AR) is a measure of the extent of cellular polarization and is defined as the ratio of length of the cell (major axis) to its width (minor axis). Solidity corresponds to cell convexity, and is derived by dividing the traced area of the cell by the area of a convex hull applied to that cell^28^. While aspect ratio corresponds to cell polarization, solidity is reflective of cell branching. Cells with a large number of processes would have low solidity while more polygonal cells would have higher solidity.

### Statistical Analysis

Statistical tests (Mann-Whitney t-test and Kruskal Wallis ANOVA for multiple comparisons) were performed using Graph Pad Prism software. D’Agostino & Pearson normality test was used to check the normality of the data distribution.

### Cell extracts and Western blotting

Lysates from glial cell cultures were prepared as previously described^6^, subjected to SDS-PAGE and blotted onto nitrocellulose. Appropriate regions of the blots were cut and incubated with specific antibodies and developed using chemiluminescent substrate (Pierce). Nuclear and cytoplasmic fractions were prepared using the Active Motif Nuclear Extract Kit (AM 40010). Briefly, for this cells were lysed using detergent treatment under hypotonic conditions and the nuclei separated by centrifugation at 14,000 g, followed by SDS-PAGE, as above. Quantitation of relative band intensity was performed using ImageJ.

### Antibodies

Antibodies used in these studies included those reactive to: MBP (Covance), anti-Rip antigen supernatant (DS Hybridoma Bank), MLC2, phosphorylated MLC2, P-(S/T) PKA substrate, lamin A/C, histone H3 (Cell Signaling Technology); laminin, phalloidin-FITC and actin (Sigma), phalloidin Acti-Stain 555 and phalloidin Acti-Stain 670 (Cytoskeleton), tubulin (Epitomics), Olig 1 and Ki-67 (Millipore), and GAPDH, lamin B and YAP/TAZ (Santa Cruz). Secondary antibodies conjugated to rhodamine, fluorescein, coumarin, or cyanin 5 were obtained from Jackson Laboratories.

## Additional Information

**How to cite this article**: Urbanski, M. *et al*. Myelinating glia differentiation is regulated by extracellular matrix elasticity. *Sci. Rep.* **6**, 33751; doi: 10.1038/srep33751 (2016).

## Supplementary Material

Supplementary Information

## Figures and Tables

**Table 1:** Measured Elastic Modulus of PA gels after polymerization.

	Acrylamide %	Bis-Acrylamide %	E± SD (kPa)
**Soft Matrix**	7.5	0.05	1.35 ± 0.09
**Rigid Matrix**	12.0	0.28	33.15 ± 2.87

This table shows the relative concentrations of acrylamide and bis-acrylamide for the soft and rigid PA gels used in these studies and their elastic modulus as measured by AFM. Four PA hydrogels per condition and 768 indentations per hydrogel (256 indentations in 3 separate 90 × 90 micron areas per gel) were assessed for these measurements.

**Table 2:** Measured Elastic Modulus of Mouse Brain and Rat Sciatic Nerves.

	*E* ± St. Dev. (kPa)
Cortex & White Matter	1.9 ± 0.9
Sciatic Nerve	
P0	6.04 ± 2.79
P2	13.77 ± 7.47
P5	24.17 ± 14.04
Adult	49.4 ± 19.0
Single teased myelinated fiber	5.3 ± 0.9

The table shows the elastic modulus values obtained from fresh coronal adult mouse brain slices and rat sciatic nerves taken during the first week after birth and adult (intact nerve and teased fiber). After removal of the surrounding fat and connective tissue, the elasticity of exposed intact nerve bundles was measured using ATF nano-indentation. A total of 3–4 sciatic nerves were measured per time point, covering an area of 90 × 90 μm/per nerve with 1024 individual measurements per area. A spherical 6.1 μm indenter was used to perform these measurements except for the single teased myelinated fiber where a 40 nm pyramidal indenter was used. For the brain slices, a spherical indenter was used (700 measurements).
