# Supplementary material for: Myelinating glia differentiation is regulated by extracellular matrix elasticity
Source: Sci Rep. 2016 Sep 20;6:33751. doi: 10.1038/srep33751 (PMC5028715; doi:10.1038/srep33751)
Supplement: Supplementary Information [file srep33751-s1.pdf]

## Supplementary Information

# Myelinating glia differentiation is regulated by extracellular matrix elasticity

Mateuz Urbanski[1,2]; Lyle Kingsbury[1];Daniel Moussouros[1]; Imran Kassim[1]; Saraf Mehjabeen[1]; Navid Paknejad[3]; Carmen V. Melendez-Vasquez[1,2*].

1. Hunter College, Department of Biological Sciences, New York, NY 10065, USA.

2. The Graduate Center, Molecular Cellular and Developmental Biology, The City University of New York, NY 10016.

3. Molecular Cytology Core Facility, Zuckerman Research Center, Memorial Sloan Kettering Cancer Center, New York, NY 10065.

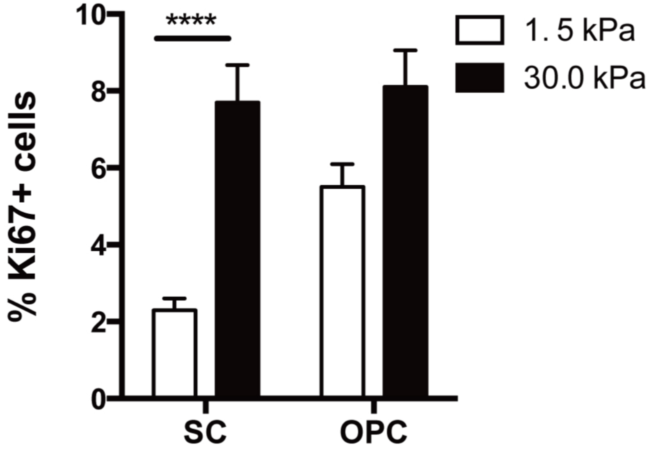


**Supplementary Figure 1**. Proliferation of Schwann cells (SC) and oligodendrocyte progenitors (OPC) in soft (1.5 kPa) and stiff (30kPa) substrates. There was a modest but significant increase (Mann Whitney t-test, $p<0.001$) in Schwann cell proliferation in stiffer matrices compared to softer ones. While OPC cultures showed the same trend, the difference in proliferation was not statistically significant (Mann Whitney t-test, $p=0.06$). Cell survival was not affected by changes in substrate stiffness and remained very low (less than 2%) as assessed by propidium iodine staining (data not shown). Data in graph represents the mean±SEM for 2 experiments (2 cultures per condition/per experiment)

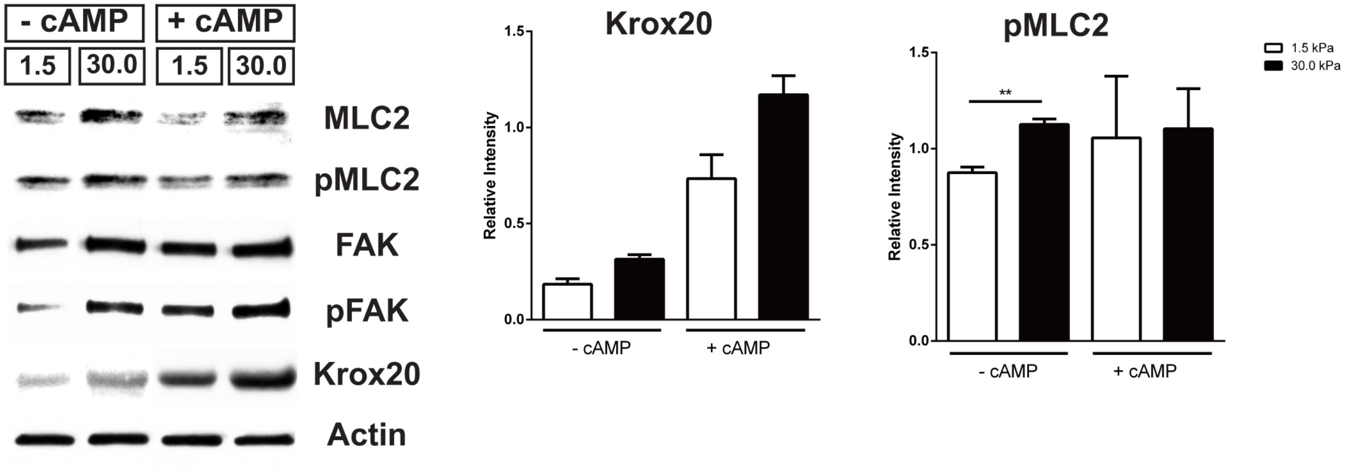


**Supplementary Figure 2**. Increased phosphorylation of the regulatory myosin light chain (pMLC2) and FAK (pFAK) were detected in SC plated in rigid matrices, indicating higher NMII activity and cell tension. These differences were abolished after addition of cAMP. Increased Krox-20 expression was observed in both soft and rigid matrices after cAMP treatment.

**A**

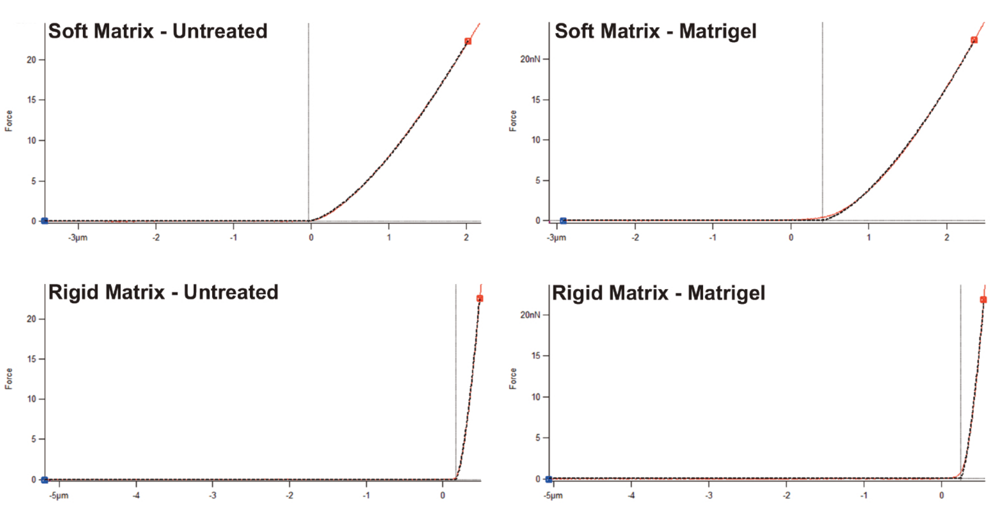


**B**

| | E ± SD (kPa) | |
|---|---|---|
| | Untreated | Matrigel Coated |
| Soft Matrix | 2.53 ± 0.26 | 2.88 ± 0.23 |
| Rigid Matrix | 44.00 ± 2.31 | 44.61 ± 5.23 |

**C**

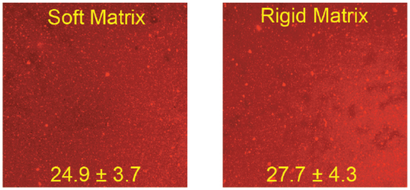


**Supplementary Figure 3**. **A**. Representative AFM force indentation curves of either uncoated substrates (left panels) or matrigel-coated substrates (right panels). The vertical dashed lines indicate the initial contact point between probe and substrate, protein coating has no observable effect on the indentation profile. **B**. Quantitation of the effects of matrigel coating on soft and rigid substrate elasticity (6 substrates tested per condition, ~ 64 measurements per substrate). Treatment with cross-linker and protein coating appear to cause a slight increase in elastic modulus, but do not significantly affect the elastic properties of the substrates. **C**. Representative images of soft and rigid matrigel-coated substrates immunostained for laminin, average fluorescence intensity per condition shown. The difference in laminin density is not statistically significant. ($p=0.22$, arbitrary units ± SD, 3 substrates stained per condition, 4 random fields imaged per substrate)
